# No sex-biased dispersal in a primate with an uncommon social system—cooperative polyandry

**DOI:** 10.7717/peerj.640

**Published:** 2014-10-30

**Authors:** Samuel L. Díaz-Muñoz, Ângela M. Ribeiro

**Affiliations:** 1 Section of Ecology, Behavior and Evolution, University of California, San Diego, La Jolla, CA, USA; 2 Department of Integrative Biology, University of California, Berkeley, CA, USA; 3 Department of Plant and Microbial Biology, University of California, Berkeley, CA, USA; 4 Interdisciplinary Centre of Marine and Environmental Research (CIIMAR/CIMAR), University of Porto, Porto, Portugal

**Keywords:** Local resource enhancement, Local mate competition, Inbreeding avoidance, Sex-biased dispersal, Population structure, Kin cooperation, Social behavior, Mating systems, Polyandry

## Abstract

An influential hypothesis proposed by Greenwood (1980) suggests that different mating systems result in female and male-biased dispersal, respectively, in birds and mammals. However, other aspects of social structure and behavior can also shape sex-biased dispersal. Although sex-specific patterns of kin cooperation are expected to affect the benefits of philopatry and dispersal patterns, empirical evidence is scarce. Unlike many mammals, *Saguinus geoffroyi* (Geoffroy’s tamarin) has a breeding system in which typically multiple males mate with a single breeding female. Males typically form cooperative reproductive partnerships between relatives, whereas females generally compete for reproductive opportunities. This system of cooperative polyandry is predicted to result in female-biased dispersal, providing an opportunity to test the current hypotheses of sex-biased dispersal. Here we test for evidence of sex-biased dispersal in *S. geoffroyi* using demographic and genetic data from three populations. We find no sex bias in natal dispersal, contrary to the prediction based on the mating patterns. This pattern was consistent after controlling for the effects of historical population structure. Limited breeding opportunities within social groups likely drive both males and females to disperse, suggesting that dispersal is intimately related to the social context. The integration of genetic and field data revealed that tamarins are another exception to the presumed pattern of male-biased dispersal in mammals. A shift in focus from mating systems to social behavior, which plays a role in most all processes expected to influence sex-bias in dispersal, will be a fruitful target for research both within species and across taxa.

*“Which sex disperses may be the outcome of a conflict between the sexes, where the relative costs and benefits of dispersal and philopatry to the sexes determine the outcome”.*

Greenwood (1980)

The factors influencing sex-specific patterns of animal dispersal have been the focus of intense research over several decades, and include competition for resources (Greenwood, 1980), competition for mates (Dobson, 1982), and inbreeding avoidance (Waser, Austad & Keane, 1986). Greenwood (1980) posited that differences in mating system, mediated by resource distributions, cause a pattern of sex-biased dispersal in mammals and birds with male and female-biased dispersal, respectively. This hypothesis, largely accepted for some time (Dobson, 2013), has now faced increased scrutiny (Dobson, 2013; Mabry et al., 2013).

Assessing the relationship between mating systems and sex-biased dispersal across taxa may be difficult because social structure and mating patterns can be variable within species. For example, red deer are strongly polygynous and expected to have “typical” mammalian male-biased dispersal. However, in different populations this pattern can disappear altogether (Perez-Espona et al., 2010) or even switch to female-biased dispersal (Pérez-González & Carranza, 2009), highlighting the importance of social structure, including changes in mate availability and group composition, in shaping sex-biased dispersal (Morelli et al., 2009).

The potential of social interactions beyond mating systems to impact dispersal patterns, although recognized for some time (Hamilton & May, 1977; Greenwood, 1980), is again an important focus of research emphasizing sex-specific patterns of kin competition and cooperation (Handley & Perrin, 2007; Clutton-Brock & Lukas, 2011; Dobson, 2013). For instance, when individuals benefit from cooperation with kin of a particular sex, there might be a selective pressure on sex-specific dispersal (Local resource enhancement model; (Perrin & Mazalov, 2000)). The potential impact of social relationships on sex-biased dispersal is illustrated in primate societies. Female primates with strong philopatry form relationships with kin that enhance fitness, as observed in female baboons (Silk et al., 2009). Similarly, philopatric male primates are observed to have strong social bonds (Di Fiore et al., 2009; Mitani, 2009). However, dispersal does not preclude cooperation between kin, as observed for male howler (Pope, 1990) and male capuchin monkeys (Jack & Fedigan, 2004; Wikberg et al., 2014), suggesting complex interactions between kin relationships and dispersal. Recent reviews (Clutton-Brock & Lukas, 2011; Dobson, 2013) have recommended the study of species with atypical mating systems, for instance mammal species in which one would expect female-biased dispersal, to test current hypotheses (Dobson, 2013). Tamarin monkeys (*Saguinus*) provide an important model because: (i) their mating patterns are predominantly polyandrous, (ii) social behavior among males tends to be cooperative, while female reproductive competition is intense and (iii) previous studies indicate that both sexes disperse (Goldizen & Terborgh, 1989; Garber et al., 1993; Lottker, Huck & Heymann, 2004; Huck, Roos & Heymann, 2007).

*Saguinus* tamarins (Callitrichinae) are Neotropical primates that typically live in groups of 3–9 individuals. The Callitrichine lineage is characterized by small body sizes, high potential reproductive output, twinning, and large neonate-maternal mass ratios; traits which are hypothesized to have co-evolved during callitrichine divergence (Harris et al., 2014). To balance the demands of infant care, tamarins form cooperatively breeding groups where multiple individuals provide alloparental care to group infants (Goldizen, 2003). Although there is considerable variation in social behavior (Goldizen, 1988), generally a single dominant breeding female mates with all adult males that are unrelated to her and enlists their assistance in caring for fraternal twin young. Tenures of breeding individuals are generally long in tamarins: 28–72 months for females, and 2–8 years for males (Garber et al., 1993; Garber, 1997; Lottker, Huck & Heymann, 2004; Díaz-Muñoz, 2011). These tenures, together with the rapid achievement of reproductive maturity (between 12 and 25 months (reviewed in Digby, Ferrari & Saltzman, 2007)), limit the breeding opportunities for other group members owing to inbreeding avoidance. The remainder of the group is typically composed of: 1–2 additional reproductive-age females, subordinates that may be daughters of the breeding female; natal adult males that delay dispersal; and 1 pair of infants or juveniles (Garber et al., 1993; Lottker, Huck & Heymann, 2004; Huck et al., 2005; Díaz-Muñoz, 2011). All group members, including subordinate adult females, provide alloparental care. However, the adult males are the primary allocare providers and group infant production correlates with the number of adult males (Garber et al., 1993). These adult males have remarkably prosocial relationships (Goldizen, 1989) and have been shown to be related and share paternity, albeit to different extents (Huck et al., 2005; Díaz-Muñoz, 2011). In contrast, female relationships are characterized by reproductive competition; the dominant female routinely suppresses subordinates in the group via behavioral and hormonal mechanisms (Savage, Ziegler & Snowdon, 1988; Garber, 1997) and as a consequence subordinates rarely produce their own offspring (Garber et al., 1993). It should be noted that tamarins have flexible social behavior and differences in ecological and social factors can lead to changes in behavior (Goldizen, 1990; Goldizen, 2003; Digby, Ferrari & Saltzman, 2007), including dispersal. Nevertheless, the properties mentioned above show remarkable consistency in studies of wild tamarins.

Given these features of tamarin social organization, i.e., local resource competition among females, kin-based cooperation among males, and inbreeding risks, we expect dispersal to be female-biased. At odds with this theoretical expectation, studies of *Saguinus* tamarins have observed dispersal by both sexes (Goldizen & Terborgh, 1989; Garber et al., 1993; Lottker, Huck & Heymann, 2004). To the best of our knowledge, only one genetic study has been conducted in *Saguinus* tamarins (Huck, Roos & Heymann, 2007) to reveal effective dispersal by both sexes in *S. mystax*; however, the study was restricted to a single population.

Thus, to robustly test the theoretical expectation of female-biased dispersal, we combined population genetic methods (using mitochondrial DNA and microsatellites) with field data from three populations of Geoffroy’s tamarin in Panama to ask:

(a) Is dispersal female biased?
(b) Is sex bias in dispersal consistent across populations?

Finally, we place these results in the larger context of the cooperative breeding system of tamarins and discuss how kin cooperation and competition may affect dispersal patterns.

## Materials and Methods

### Geographic location and individual dispersal status

**Figure 1:**
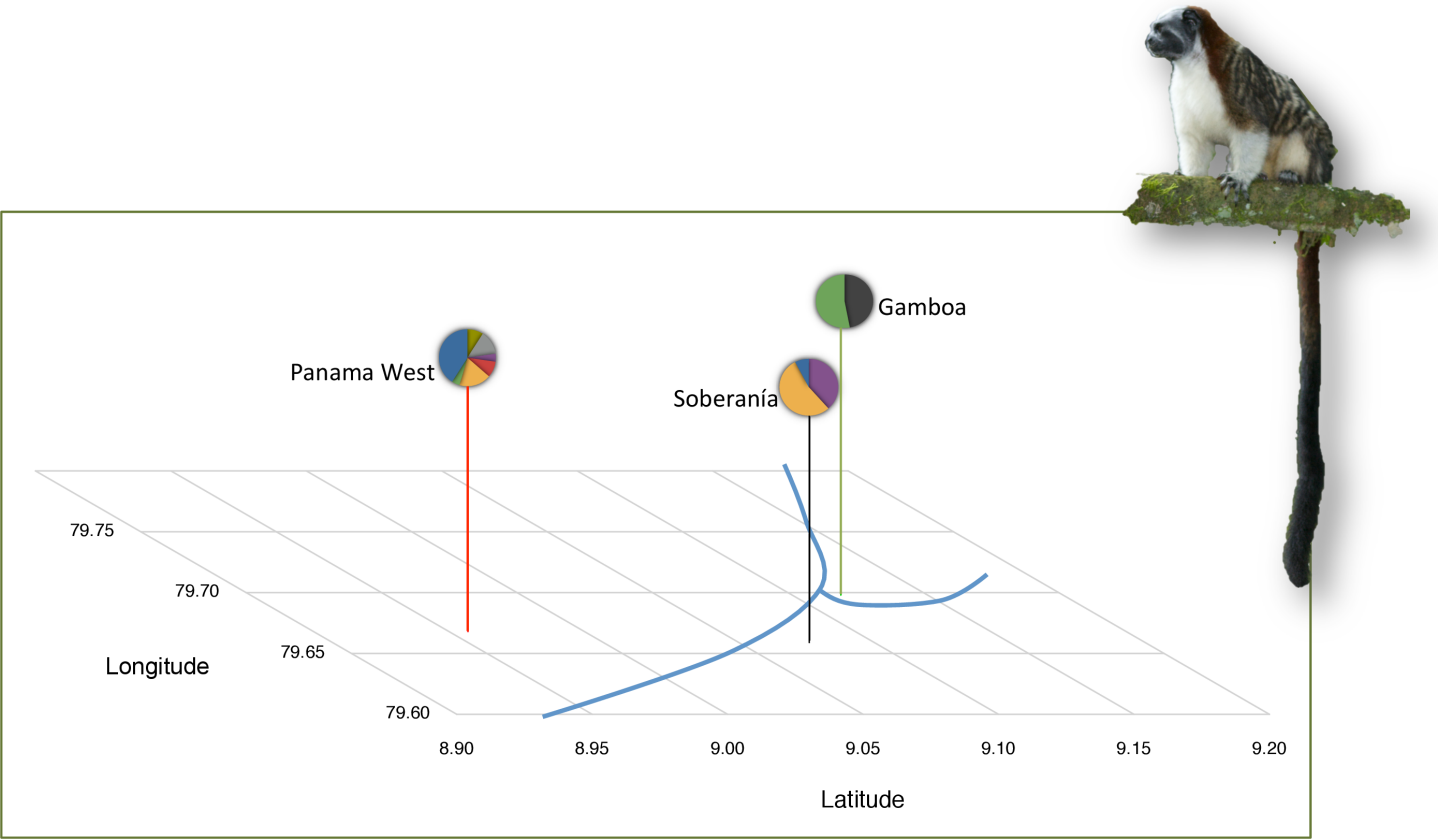
Background information on tamarin study populations. Population locations in Panama Canal watershed and mitochondrial lineages are shown per Díaz-Muñoz (2012). Mitochondrial lineages as represented by distinct colors in the pie charts, where each color depicts a different mtDNA haplotype. An exact test of population divergence supports the presence of two distinct groups. Blue lines depict riverine water barriers: Panama Canal and Chagres River. Photo Credit: Anand Varma.

We sampled 44 tamarins in three localities in Panama (Fig. 1): Gamboa, Panama West, and Soberania National Park. The number of social groups and individuals in each locality are detailed in Table 1. Data on weight, age, and sex were recorded in the field. For the Soberanía and Gamboa populations, we used behavioral observations and long-term demographic data to ascertain dispersal status. Individuals that were associated with a lactating female and with mass <400 gr at capture were considered infants born into the group; older offspring not associated with a lactating female were considered juveniles. Individuals >400 gr were considered adults. The Panama West individuals were sampled from museum specimens collected by GA Dawson (Michigan State University Museum (Díaz-Muñoz, 2012)) and no behavioral or group composition information was available, except that individuals sampled represent multiple groups and no complete groups. Thus, dispersal status was assigned on the basis of body mass. Full geographic locations and field methods are described elsewhere (Díaz-Muñoz, 2011; Díaz-Muñoz, 2012). This research project was authorized by the National Authority of the Environment of the Republic of Panama (SE/A-17-05, SE/A-16-06, SE/A-13-07, SE/A-10-08, SE/A-6-09) and import of samples under CITES was authorized by the US Fish and Wildlife Service (09US224310/9). The animal handling and use procedures in this project were approved by the UC Berkeley Institutional Animal Care and Use Committee (MAP #R224-030) and followed the published guidelines for animal use of the American Society of Mammalogists (Gannon & Sikes, 2007) and the ASAB/ABS (2006) guidelines.

**Table 1:** Population sample demographics. Number of social groups and individuals in each of the Geoffroy’s tamarin populations studied.

Populations	Gamboa (*n* = 17)			Soberania (*n* = 14)			Panama West (*n* = 13)
Groups	BA	LC	PH	NJ	CT^b^	AG^b^	Unknown^a^
Individuals	3	7	7	6	7	6	13
Adult males	2	3	4	2	2	2	5
Adult females	1	1	1	2	1	1	5
Infants/Juveniles	0	3	3	4	2	2	
Total adults		12			8		10
Total Ind. <400 g		5			6		3

**Notes.**

a Individuals sampled from museum specimens and group compositions were not available, but samples do not represent complete groups and were collected from multiple groups.

b Groups CT and AG not sampled completely, missing 2 and 1 adults respectively. Only the total reflects uncaptured individuals.

### Genetic analyses

We used seven microsatellite loci to obtain individual genotypes with no missing data (Díaz-Muñoz, 2011). Procedures for DNA extraction, amplification, and scoring have been reported in detail previously (Díaz-Muñoz, 2011). In brief, we extracted DNA from hair or tissues using Qiagen DNA Micro kits (Qiagen, Valencia, CA). For Panama West samples (museum specimens) we used a dedicated room. Following PCR amplification, we genotyped samples in an ABI 3730 automated sequencer (ABI, Foster City, CA) and scored in Genemapper 4.0 (ABI).

No microsatellites deviated from Hardy–Weinberg expectations or showed linkage disequilibrium. We employed a two-step approach to examine genetic structure and infer sex-biased dispersal:

**1. *Compare male and female groups***. We compared male and female population genetic structure in three populations using three distinct statistics that allow for independent tests within site, without relying on between-site comparisons: *Fis* (Wright, 1942), *AIc* (assignment index corrected by population mean (Paetkau et al., 1995)) and *R* (relatedness; (Ritland, 1996)).

Under sex-biased dispersal the pool of genotypes of the dispersing sex will exhibit a deficiency of heterozygotes caused by immigrant genotypes. If dispersal is female-biased, we expect *Fis* for females to be positive and larger than *Fis* for males. Thus, we tested for female-biased dispersal estimating *Fis* in Genepop (Rousset, 2008) by setting the alternative hypothesis to heterozygote deficiency.

Individuals living in close proximity are expected to be more related than individuals taken at random from the whole population. Therefore, we calculated average pairwise relatedness, *R*, for each sex within each population using GenAlEx v6.5 (Peakall & Smouse, 2012). As per our prediction of female-biased dispersal, we tested whether the philopatric sex (males) had higher average relatedness than the dispersing sex (females) using a Wilcoxon test.

With the rationale that immigrants (dispersers) can introduce new alleles into the population, we estimate the probability that an individual is a resident or an immigrant using *AIc* (calculated in GenAlEx v6.5 Peakall & Smouse, 2012). *AIc* is centered on zero; positive values characterize individuals with a higher probability of being ‘residents’, whereas negative values indicate a high probability of being ‘immigrants’. We tested the hypothesis that female-biased dispersal would create negative *AIc* values due to immigration (i.e., females are mostly immigrants) by using a Wilcoxon test to detect significant differences in *AIc* between the sexes.

**2. *Compare pre-dispersal with post-dispersal groups***. We pooled genotypes from all three populations to build two groups: (i) infants and juveniles (*pre-dispersal* individuals *n* = 14) and (ii) adults (*post-dispersal* individuals *n* = 30). Defining a *pre-dispersal* group (i.e., infant/juveniles) allowed us to build a null control for sex-biased dispersal. Specifically, because infants and juveniles represent a random assortment of alleles yet to disperse, any sex-specific pattern of relatedness and population structure that might exist in the population should be erased. Therefore, if dispersal is female-biased in tamarins we expect: adult females to be less related than under a scenario of unbiased dispersal; and significant difference in mean *AIc* between adult males and females, but no difference between young/juvenile males and females (*pre-dispersal* group). To test these predictions, we first compared the observed *R* to a null distribution obtained by randomization of infant/juvenile or adult genotypes (10,000×) and tested whether it exceeded the 95% CI. Second, we calculated the difference in the *AIc* mean (*D* = |*AIc females*−*AIc males*|) and built a null distribution by randomization. We tested whether observed *D* for each group (i.e., infant/juvenile and adults) was significantly different from a null distribution of *D*, generated by randomly assigning sex to individuals 9,999 times. Probability of obtaining a result that exceeded the null hypothesis was *p* = [(number *D null* ≥ *D*)/total number randomizations].

These approaches avoid common statistical pitfalls (Prugnolle & de Meeus, 2002), by using the same bi-parentally inherited loci across populations and randomizing sex among individuals in each population (Prugnolle & de Meeus, 2002). Finally, because the 44 genotypes used were sampled across a broad geographic area (∼500 km^2^) and previous work demonstrated population divergence in this area (Díaz-Muñoz, 2012), we controlled for the possible effect of population structure. We used an ANCOVA with *AIc* mean as the dependent variable and the result from an exact test of population divergence based on 1,080 base pairs of the mitochondrial control region (i.e., presence of two genetically distinct groups; see Fig. 1) as the covariate. In this manner we could distinguish variation in *AIc* reflecting historical changes from variation in sex-specific *AIc*. Statistical analyses were performed using R v3.0.0 (R Development Core Team, 2013).

## Results and Discussion

**Figure 2:**
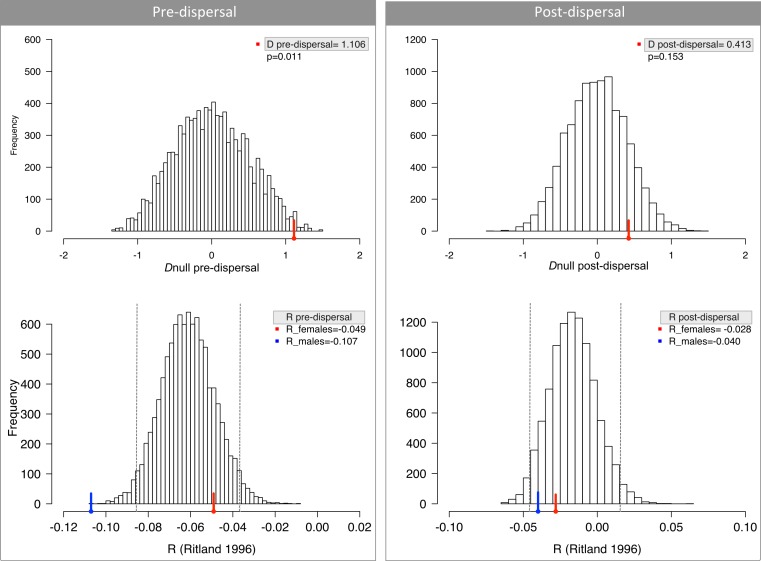
No difference in dispersal between males and females measured by relatedness (*R*) and difference in assignment index (D). Pre-dispersal group consists of young and juveniles and hence is a random assortment of alleles from all parents. Post-dispersal group comprises exclusively adults and therefore represents the populations after the movement of alleles due to dispersal. Dashed lines depict the 95% CI around mean *R*.

The genetic analyses presented suggest that there is no sex bias in effective dispersal in *S. geoffroyi*, contrary to our initial prediction (Table 2 and Fig. 2). *Fis* for females was consistently negative, at odds with the expectation of a positive *Fis* for the dispersing sex. *R*-values were similar between sexes and *AIc* suggests no sex-differential presence of immigrants over residents (Table 2). Interestingly, this pattern held across three populations and is consistent with previous observations for *Saguinus mystax* (Huck, Roos & Heymann, 2007). Our results were not confounded by historical genetic structure: there was no statistically significant difference between sexes in *AIc* (ANCOVA *F*_1,30_ = 1.270, *p* = 0.270), when analyzing only adults. This suggests the dispersal pattern is consistent across space, at least in this focal area. Although not systematically examined here, this pattern may also be stable across time as one population was sampled from museum specimens. Temporal differences in sex-biased dispersal may be important, particularly for species undergoing rapid anthropogenic change (Perez-Espona et al., 2010).

**Table 2:** Dispersal is not sex-biased in Geoffroy’s tamarins. Predictions under female-biased dispersal for statistics and tests are indicated in light shading. Gamboa, Panama West, Soberania: per-population results. Pre-dispersal and Post-dispersal: infants/juveniles (pre-) vs. adult (post-) results.

			*Fis*	*R*	*AIc mean*	*AIc var*
	Prediction		*F* > *M*	*F* < *M*	*F* < *M*	*F* > *M*
**Gamboa**		Males (*n* = 10)	−0.281 (*p* = 0.999)	−0.041	−0.072	0.630
		Females (*n* = 7)	−0.378 (*p* = 0.999)	−0.057	0.103	0.818
					W; *p* = 0.962	
**Panama West**		Males (*n* = 7)	−0.091 (*p* = 0.762)	−0.072	0.189	0.209
		Females (*n* = 6)	−0.100 (*p* = 0.733)	−0.086	−0.220	1.912
					W; *p* = 0.945	
**Soberania**		Males (*n* = 6)	−0.236 (*p* = 0.231)	−0.092	−0.056	0.337
		Females (*n* = 8)	−0.236 (*p* = 0.981)	−0.058	0.042	1.004
					W; *p* = 0.485	
	Prediction		*F* = *M*	*F* = *M*	*F* = *M*	*F* = *M*
**Pre-dispersal**		Males (*n* = 5)	−0.057	−0.107	−0.712	0.749
		Females (*n* = 9)	−0.354	−0.049	0.395	0.287
					ANCOVA: 0.017	
	Prediction		*F* > *M*	*F* < *M*	*F* < *M*	*F* > *M*
**Post-dispersal**		Males (*n* = 18)	−0.053	−0.040	0.112	0.706
		Females (*n* = 12)	−0.152	−0.028	−0.167	1.662
					ANCOVA: 0.457	

**Notes.**

*Fis* Wright’s F statistic *R* Ritland’s relatedness *AIc* Peatkau’s assignment index corrected by population mean ANCOVA (analysis of covariance) W Wilcoxon test

We did find significant differences in *R* and *D* for infants/juveniles (*pre-dispersal*), despite our prediction of similar values between sexes (Fig. 2); and after accounting for historical genetic structure *AIc* values were still significant (ANCOVA; Table 2). A closer inspection of the genotypes revealed that our estimates were biased because some juvenile males had unique alleles in the population. This result may be explained by the stochasticity associated with sampling procedure and the sample sizes; or alternatively, these juveniles were mis-assigned as *pre-dispersal* by our criteria and are indeed represented tamarins that had already dispersed. Nevertheless, all other analyses, notably those that do not depend on identifying pre- and post-dispersal groups, suggest that there is no sex bias in effective dispersal in these Geoffroy’s tamarin populations.

The absence of sex bias in dispersal seems counterintuitive in light of the resource defense hypothesis, where male mammals benefit from philopatry (Perrin & Mazalov, 2000). If, in general, females are reproductive competitors and males typically cooperate in kin groups, why would both sexes disperse? Cooperation and competition can also occur *within* a sex, especially in cooperative breeders: while *S. geoffroyi* females are typically competitive in regard to reproduction, subordinate females often assist in infant rearing and may also be related to breeding females. Adult males in a group are generally related and cooperate in reproductive contexts, but these two properties need not be related exclusively to philopatry; related male tamarins often disperse together or reunite in new groups (Lottker, Huck & Heymann, 2004). Although cooperation may provide benefits to philopatry (local resource enhancement hypothesis), the monopoly of the breeding female simultaneously restricts breeding opportunities for both sexes (Garber, 1997). When breeding tenures of the dominant females are long, as is generally the case for tamarins (Goldizen & Terborgh, 1989; Garber et al., 1993; Goldizen et al., 1996; Savage et al., 1996), breeding opportunities for natal group members are scarce or absent, if individuals are to avoid mating with kin. As a consequence, individuals of both sexes are expected to disperse to seek breeding opportunities.

The ability to detect sex-biased dispersal genetically depends crucially on the power of the tests employed and the characteristics of dispersal such as rate and distance. Many genetic tests are insensitive to asymmetrical dispersal between genders unless the bias is extreme (Goudet, Perrin & Waser, 2002) and a large sample size is analyzed. We employed several measures to ensure our genetic tests avoided common statistical pitfalls. Specifically we used the same bi-parentally inherited loci across populations, randomized sex among individuals within each population, and included only same-season samples within populations (Prugnolle & de Meeus, 2002). We cannot exclude the possibility that a small bias in philopatry was not detected in our study due to a lack of statistical power. However, our results suggest this is likely not the case. The observed heterozygosity for each population (*H*_*O* Gamboa_ = 0.7924; *H*_*O* Panama West_ = 0.675; *H*_O Soberania_ = 0.630) was large suggesting high levels of gene flow. Thus, if rates of dispersal were highly skewed toward one sex, the tests we implemented would be able to detect the signal. Moreover, the reported lack of sex-biased dispersal, adds to existing demographic (Goldizen & Terborgh, 1989; Lottker, Huck & Heymann, 2004) and genetic (Huck et al., 2005) evidence suggesting both sexes disperse, in *Saguinus*. One further question that remains to be elucidated is dispersal distance. Evidence from *S. mystax* suggests that females may move longer distances (Huck, Roos & Heymann, 2007), perhaps due to limited reproductive opportunities associated with male-biased groups with males sharing reproduction with a single breeding female. However, dispersal by both sexes may imply fitness costs that arise from kin competition or the risk of inbreeding. Only future detailed studies integrating demography, behavior, and genetics over the long term will help illuminate the factors associated with sex-specific dispersal patterns.

In sum, the analyses suggest that sex-unbiased dispersal is a robust property of *S. geoffroyi* likely arising from its social organization. In social animals, limited breeding opportunities within groups set the stage for conflicts of interest in reproduction, resulting in a mating system that can be viewed as an emergent property of social interactions (Davies et al., 1995). While within-sex cooperation is an important selective pressure for philopatry, in *S. geoffroyi* this occurs in both males and females and thus the risk of philopatric inbreeding needs to be compensated by dispersing, regardless of sex.

Our empirical study reveals that local resource enhancement might be as important as local resource competition and inbreeding avoidance in determining the evolution of dispersal (Perrin & Mazalov, 2000).

## Additional Information and Declarations

### Field Study Permissions

The following information was supplied relating to field study approvals (i.e., approving body and any reference numbers): This research project was authorized by the National Authority of the Environment of the Republic of Panama (SE/A-17-05, SE/A-16-06, SE/A-13-07, SE/A-10-08, SE/A-6-09) and import of samples under CITES was authorized by the US Fish and Wildlife Service (09US224310/9).

### Data Deposition

The following information was supplied regarding the deposition of related data: Data and code for analyses are deposited in Figshare: DOI 10.6084/m9.figshare.1209605.

## References

[ref-1] Clutton-Brock TH, Lukas D (2011). The evolution of social philopatry and dispersal in female mammals. Molecular Ecology.

[ref-2] Davies NB, Hartley IR, Hatchwell BJ, Desrochers A, Skeer J, Nebel D (1995). The polygynandrous mating system of the alpine accentor, *Prunella collaris*. I. Ecological causes and reproductive conflicts. Animal Behavior.

[ref-3] Di Fiore A, Link A, Schmitt CA, Spehar SN (2009). Dispersal patterns in sympatric woolly and spider monkeys: integrating molecular and observational data. Behaviour.

[ref-4] Díaz-Muñoz SL (2011). Paternity and relatedness in a polyandrous nonhuman primate: testing adaptive hypotheses of male reproductive cooperation. Animal Behavior.

[ref-5] Díaz-Muñoz SL (2012). Role of recent and old riverine barriers in fine-scale population genetic structure of Geoffroy’s tamarin (*Saguinus geoffroyi*) in the Panama Canal watershed. Ecology and Evolution.

[ref-6] Digby LJ, Ferrari SF, Saltzman W, Campbell CJ, Fuentes AF, Mackinnon KC, Panger M, Bearder S (2007). Callitrichines: the role of competition in cooperatively breeding species. Primates in perspective.

[ref-7] Dobson FS (1982). Competition for mates and predominant juvenile male dispersal in mammals. Animal Behavior.

[ref-8] Dobson FS (2013). The enduring question of sex-biased dispersal: Paul J. Greenwood’s (1980) seminal contribution. Animal Behavior.

[ref-9] Gannon W, Sikes R (2007). Guidelines of the American Society of mammalogists for the use of wild mammals in research. Journal of Mammalogy.

[ref-10] Garber PA (1997). One for all and breeding for one: cooperation and competition as a tamarin reproductive strategy. Evolutionary Anthropology: Issues, News, and Reviews.

[ref-11] Garber PA, Encarnación F, Moya L, Pruetz JD (1993). Demographic and reproductive patterns in moustached tamarin monkeys (*Saguinus mystax*): implications for reconstructing platyrrhine mating systems. American Journal of Primatology.

[ref-12] Goldizen AW (1988). Tamarin and marmoset mating systems: unusual flexibility. Trends in Ecology and Evolution.

[ref-13] Goldizen AW (1989). Social relationships in a cooperatively polyandrous group of tamarins (*Saguinus fuscicollis*). Behavioral Ecology and Sociobiology.

[ref-14] Goldizen A (1990). A comparative perspective on the evolution of tamarin and marmoset social systems. International Journal of Primatology.

[ref-15] Goldizen AW, Reichard UH, Boesch C (2003). Social monogamy and its variations in callitrichids do these relate to the costs of infant care. Monogamy: mating strategies and partnerships in birds, humans and other mammals.

[ref-16] Goldizen AW, Mendelson J, Van Vlaardingen M, Terborgh J (1996). Saddle-back tamarin (*Saguinus fuscicollis*) reproductive strategies: evidence from a thirteen-year study of a marked population. American Journal of Primatology.

[ref-17] Goldizen AW, Terborgh J (1989). Demography and dispersal patterns of a tamarin population: possible causes of delayed breeding. The American Naturalist.

[ref-18] Goudet J, Perrin N, Waser P (2002). Tests for sex-biased dispersal using bi-parentally inherited genetic markers. Molecular Ecology.

[ref-19] Greenwood PJ (1980). Mating systems, philopatry and dispersal in birds and mammals. Animal Behavior.

[ref-20] Hamilton WD, May RM (1977). Dispersal in stable habitats. Nature.

[ref-21] Handley LJ, Perrin N (2007). Advances in our understanding of mammalian sex-biased dispersal. Molecular Ecology.

[ref-22] Harris RA, Tardif SD, Vinar T, Wildman DE, Rutherford JN, Rogers J, Worley KC, Aagaard KM (2014). Evolutionary genetics and implications of small size and twinning in callitrichine primates. Proceedings of the National Academy of Sciences of the United States of America.

[ref-23] Huck M, Löttker P, Böhle U-R, Heymann EW (2005). Paternity and kinship patterns in polyandrous moustached tamarins (*Saguinus mystax*). American Journal of Physical Anthropology.

[ref-24] Huck M, Roos C, Heymann EW (2007). Spatio-genetic population structure in mustached tamarins, *Saguinus mystax*. American Journal of Physical Anthropology.

[ref-25] Jack KM, Fedigan L (2004). Male dispersal patterns in white-faced capuchins, *Cebus capucinus*: Part 2: patterns and causes of secondary dispersal. Animal Behavior.

[ref-26] Lottker P, Huck M, Heymann E (2004). Demographic parameters and events in wild moustached tamarins (*Saguinus mystax*). American Journal of Primatology.

[ref-27] Mabry KE, Shelley EL, Davis KE, Blumstein DT, Van Vuren DH (2013). Social mating system and sex-biased dispersal in mammals and birds: a phylogenetic analysis. PLoS ONE.

[ref-28] Mitani JC (2009). Male chimpanzees form enduring and equitable social bonds. Animal Behavior.

[ref-29] Morelli TL, Wright P, King S, Pochron S (2009). The rules of disengagement: takeovers, infanticide, and dispersal in a rainforest lemur, *Propithecus edwardsi*. Behaviour.

[ref-30] Paetkau D, Calvert W, Stirling I, Strobeck C (1995). Microsatellite analysis of population structure in Canadian polar bears. Molecular Ecology.

[ref-31] Peakall R, Smouse PE (2012). GenAlEx 6.5: genetic analysis in Excel. Population genetic software for teaching and research–an update. Bioinformatics.

[ref-32] Perez-Espona S, Perez-Barberia FJ, Jiggins CD, Gordon IJ, Pemberton JM (2010). Variable extent of sex-biased dispersal in a strongly polygynous mammal. Molecular Ecology.

[ref-33] Pérez-González J, Carranza J (2009). Female-biased dispersal under conditions of low male mating competition in a polygynous mammal. Molecular Ecology.

[ref-34] Perrin N, Mazalov V (2000). Local competition, inbreeding, and the evolution of sex-biased dispersal. The American Naturalist.

[ref-35] Pope TR (1990). The reproductive consequences of male cooperation in the red howler monkey: paternity exclusion in multi-male and single-male troops using genetic markers. Behavioral Ecology and Sociobiology.

[ref-36] Prugnolle F, de Meeus T (2002). Inferring sex-biased dispersal from population genetic tools: a review. Heredity.

[ref-37] R Development Core Team (2013). R: a language and environment for statistical computing.

[ref-38] Ritland K (1996). Estimators for pairwise relatedness and individual inbreeding coefficients. Genetical Research.

[ref-39] Rousset F (2008). genepop’007: a complete re-implementation of the genepop software for Windows and Linux. Molecular Ecology Resources.

[ref-40] Savage A, Giraldo LH, Soto LH, Snowdon CT (1996). Demography, group composition, and dispersal in wild cotton-top tamarin (*Saguinus oedipus*) groups. American Journal of Primatology.

[ref-41] Savage A, Ziegler TE, Snowdon CT (1988). Sociosexual development, pair bond formation, and mechanisms of fertility suppression in female cotton-top tamarins (*Saguinus oedipus*). American Journal of Primatology.

[ref-42] Silk JB, Beehner JC, Bergman TJ, Crockford C, Engh AL, Moscovice LR, Wittig RM, Seyfarth RM, Cheney DL (2009). The benefits of social capital: close social bonds among female baboons enhance offspring survival. Proceedings of the Royal Society B.

[ref-43] Waser PM, Austad SN, Keane B (1986). When should animals tolerate inbreeding?. The American Naturalist.

[ref-44] Wikberg EC, Jack KM, Campos FA, Fedigan LM, Sato A (2014). The effect of male parallel dispersal on the kin composition of groups in white-faced capuchins. Animal Behavior.

[ref-45] Wright S (1942). Statistical genetics and evolution. Bulletin of the American Mathematical Society.

